# Longitudinal Associations Between Timing of Physical Activity Accumulation and Health: Application of Functional Data Methods

**DOI:** 10.1007/s12561-022-09359-1

**Published:** 2022-09-29

**Authors:** Wenyi Lin, Jingjing Zou, Chongzhi Di, Dorothy D. Sears, Cheryl L. Rock, Loki Natarajan

**Affiliations:** 1 Division of Biostatistics, Herbert Wertheim School of Public Health and Longevity Science, University of California, San Diego, La Jolla, CA, USA; 2 Public Health Sciences Division, Fred Hutchinson Cancer Research Center, Seattle, WA, USA; 3 College of Health Solutions, Arizona State University, Phoenix, AZ, USA; 4 Department of Medicine, University of California, San Diego, La Jolla, CA, USA; 5 Department of Family Medicine, University of California, San Diego, La Jolla, CA, USA

**Keywords:** Accelerometer, Physical activity, Functional modeling, Principal component analysis, Longitudinal data analysis

## Abstract

Accelerometers are widely used for tracking human movement and provide minute-level (or even 30 Hz level) physical activity (PA) records for detailed analysis. Instead of using day-level summary statistics to assess these densely sampled inputs, we implement functional principal component analysis (FPCA) approaches to study the temporal patterns of PA data from 245 overweight/obese women at three visits over a 1-year period. We apply longitudinal FPCA to decompose PA inputs, incorporating subject-specific variability, and then test the association between these patterns and obesity-related health outcomes by multiple mixed effect regression models. With the proposed methods, the longitudinal patterns in both densely sampled inputs and scalar outcomes are investigated and connected. The results show that the health outcomes are strongly associated with PA variation, in both subject and visit-level. In addition, we reveal that timing of PA during the day can impact changes in outcomes, a finding that would not be possible with day-level PA summaries. Thus, our findings imply that the use of longitudinal FPCA can elucidate temporal patterns of multiple levels of PA inputs. Furthermore, the exploration of the relationship between PA patterns and health outcomes can be useful for establishing weight-loss guidelines.

## 1 Introduction

Physical inactivity and sedentary behavior are known risk factors for cardiovascular disease (CVD), cancer and mortality [3, 7, 21, 24]. Increased physical activity has been demonstrated to improve cardiopulmonary fitness and promote healthy weight management [22]. Current CDC guidelines recommend engaging in 150 min/week or more of moderate-vigorous-activity in order to maintain a healthy weight, and for reducing the risk of hypertension, diabetes, heart attacks, and stroke, as well as, osteoporosis, risk of falls, and depression [9]. Given the multitude of health benefits, it is important to develop robust and informative statistical models for exploring the relationship between all aspects of physical activity (PA) and health outcomes.

Traditional approaches for collecting information about an individual’s PA have relied heavily on self-reported questionnaires, sleep-logs, and daily diaries [30]. However, these methods require an individual to recall their PA over a previous period, and hence are often inaccurate and/or biased. Further, these methods do not usually obtain daily PA level or elicit information regarding PA accumulation patterns throughout the day [1]. Because accurate and consistent measurement of PA is critical for designing and assessing interventions, devices such as accelerometers, are increasingly used for recording objective estimates of PA [18]. These devices are self-worn sensors and measure PA based on activity counts derived from high-resolution acceleration signals obtained at the minute-level, or even second level.

Most studies utilizing accelerometers have focused on aggregate or summary statistics such as daily total or weekly average activity counts or minutes of moderate-vigorous physical activity (MVPA) [32]. While such summary measures of activity are easy to understand and implement using standard statistical techniques, aggregating activity records to daily or weekly averages results in a loss of information. In particular, summarizing precludes the evaluation of temporal variation (e.g., the timing during the day) of PA, which may provide additional insight into associations between diurnal variation in PA and health outcomes.

Functional data analysis is a powerful and well-studied statistical method [25, 31] for modeling curves or functions that are continuous. In the context of PA, functional data methods, and functional principal components analysis (FPCA) in particular, can better elucidate patterns of the full spectrum of accelerometer data. In essence, this approach treats each participant’s activity profile as a single functional datum, rather than reducing it to a scalar summary. Various models have been developed to explore the minute-level information, extrapolating from densely sampled accelerometer inputs rather than simply implementing daily or weekly summaries [8, 12, 17, 23, 25, 29]. The review paper by Ramsay et al. [25], provides an overview of methods and applications in FPCA. The main idea is to decompose the dense signal inputs and to extract the principal variation directions, thus reducing the dimension. The FPCA searches for a set of mutually orthogonal and normalized weight functions to summarize subject-specific features. This idea was generalized to multilevel FPCA, which captures both the intra- and inter-subject variation [12]. In addition, Greven et al. [17] proposed longitudinal FPCA to include dynamic subject-specific variability and Shou et al. [29] extended the analysis to decompose the variability of any functional model with a particular linear structure via structured FPCA. Due to the hierarchical structure of our data with repeated days clustered within subjects and visits, longitudinal FPCA is implemented in this paper in order to obtain information from the entire accelerometer signal inputs, while at the same time accounting for the nested structure of our data.

Much statistical research has focused on developing regression models to evaluate associations between these functional measures of activity and health outcomes. Crainiceanu et al. [11] proposed a framework for regression models where the functional predictor is repeatedly observed but the response is a scalar variable. Along these lines, our previous study [35] implemented a multilevel FPCA to characterize subject- and visit-level variation, and used the corresponding principal component scores as predictors to examine associations between PA patterns and health outcomes. Similarly, several studies have utilized functional data methods to investigate accelerometer-measured physical activity and health [2, 6, 28], but these studies have been primarily cross-sectional, and/or the methods do not apply to longitudinally collected exposures (i.e., physical activity) and health outcomes, which is a focus of prospective epidemiologic studies. There have been methodological advances in the statistical realm. To model the longitudinal structure, Goldsmith et al. [16] extended the spline-based estimation strategy on functional predictors and added subject-specific random effects to the standard cross-sectional setting. Furthermore, combined with longitudinal FPCA [17], Gertheiss et al. [15] were able to incorporate the longitudinal structure of the functional predictors in the regression model. Thus, these models include subject-specific effects and functional predictors in the regression model, and can be summarized as functional mixed effect models.

In this paper, extending our earlier cross-sectional investigation [35], we implemented longitudinal FPCA and functional mixed effects models to investigate associations between diurnal PA patterns and longitudinal health outcomes. To this end, we leveraged data from a dietary intervention weight-loss trial of 245 overweight women (the MENU Study [20, 26]) with accelerometry and a wide array of glucoregulatory and inflammatory biomarkers collected at three visits over 12 months. We used a two-step approach. In the first step, a longitudinal FPCA was applied to incorporate subject- and visit-specific variability when decomposing functional inputs. In the second step, mixed effect models were fitted with functional predictors from the first step to inform the association between PA and health outcomes. By applying this procedure, we not only addressed the subject-to-subject and visit-to-visit variation in activity patterns, but also made more nuanced inferences about how diurnal patterns of physical activity could longitudinally affect weight-loss and biomarkers related to obesity.

## 2 Study Overview

The MENU weight-loss study (2011–2017) [20, 26], a project in the UC San Diego NIH-funded Transdisciplinary Research on Energetics and Cancer (TREC) Center, comprised 245 non-diabetic and overweight/obese women. Participants were randomized to one of three diet arms for investigating how variation in macronutrient diet composition impacted weight-loss and cardiometabolic biomarkers. All participants across the diet arms also received a physical activity intervention. Eligibility criteria for study participation were age ≥ 21 years, body mass index (BMI; kg/m^2^) between 27 and 40, and willingness and ability to participate in clinic visits, group sessions, and telephone and internet communications during the 12-month study.

Clinic visits, measurements and data collection occurred at three time-points: baseline and 6 and 12 months. Demographic data, including age, ethnicity and smoking status, were collected only at the baseline visit. Fasting levels of C-reactive protein (CRP), insulin and body mass index (BMI) were measured at each visit and these constituted the longitudinal (scalar) health outcomes in our analysis. In general, larger values of each outcome indicate worse health status. Insulin and CRP were log-transformed so that the distribution of the transformed data are close to Gaussian. Details of the study protocol and main results have been previously published [20, 26].

Physical activity (PA), measured with accelerometer devices GT3X Actigraph (ActiGraph, LLC; Pensacola, FL), was recorded daily at each visit. The devices collect acceleration data at 30 Hz on the x, y, z axes and then the ActiLife program applies a band-pass filter to remove non-human acceleration signals from the data. The triaxial activity counts vector $\left({\text{AC}}_{x},{\text{AC}}_{y},{\text{AC}}_{z}\right)$ are summarized as magnitudes $\sqrt{{\text{AC}}_{x}^{2}+{\text{AC}}_{y}^{2}+{\text{AC}}_{z}^{2}}$, which are referred to as activity magnitude in the manuscript and related to intensity of the activity [4]. These activity counts can be categorized into minutes spent in sedentary, light, moderate, and vigorous activity using calibration thresholds. Participants were instructed to wear the devices for 7 days during waking hours, except when in contact with water. Non-wear time was identified via pre-defined algorithms of consecutive zero counts using standard protocols [10] and labeled as missing data. Records with at least 10 h of device wear (per standard protocols) were retained. The final dataset includes accelerometer data for 4259 days for 245 participants; 4 records with fewer than 10 h of wear were removed. All participants received the same physical activity intervention regardless of diet group. Thus for the current investigation the three diet arms are combined and the study is analyzed as a longitudinal cohort.

## 3 Statistical Model

### 3.1 Accelerometer Data Processing

**Fig. 1:**
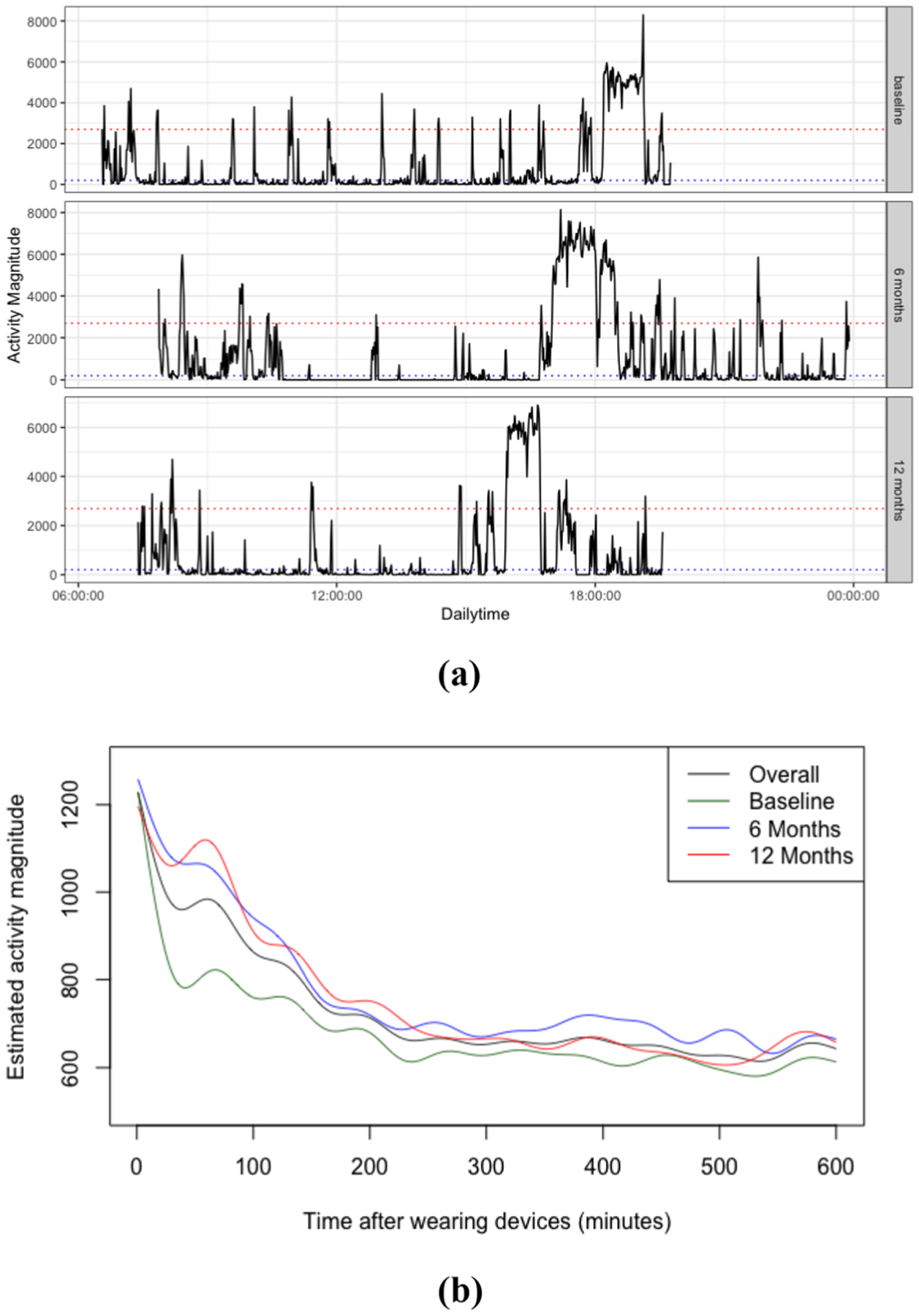
The plots provide **a** an example of activity patterns from minute-level accelerometer count data for one subject across three visits: the raw activity curve (black solid line), the sedentary count threshold (blue dotted line) and the MVPA count threshold (red dotted line); **b** the smoothed overall and visit-level mean activity magnitude curves at baseline, 6 months and 12 months. The y-axis denotes estimated activity magnitude and the x-axis depicts a time sequence from the start of devices wear (0) up to 600 min

We proposed analysis models based on the PA time-series inputs. Figure 1 presents an example of activity records from 6:00 am to 11:29 pm for one participant on the first day of each visit. Each data point (y-axis) represents minute-wise PA activity magnitude. Based on calibration studies on energy expenditure, sedentary time is defined as minutes with activity magnitude < 200, and moderate to vigorous physical activity (MVPA) time is defined as minutes with activity magnitude > 2690 [27].

As shown in Fig. 1a, the starting time and duration time are not constant for a given participant across days, and furthermore, these measures also vary among participants. To be specific, the mean time for participants to start wearing the device was around 7 am (SD 127 min), indicating that participants, generally started daily activities in the morning. Therefore, to ensure a more consistent and balanced data structure, we re-aligned daily records, so that all participants had a “common” starting time of device wear denoted as “0” on the x-axis in subsequent plots, so that 10 h of device wear are recorded as 0 to 600 min (10 h) on the x-axis. This realignment ensures that the start and end times across all days and participant activity profiles are on the same grid of points.

Lastly, the daily activity data for each participant were averaged over days within each visit to obtain an averaged PA profile for each visit. Of note, the mean (SD) number of days of device data per participant was 3.9 (SD 2.1). Sensitivity analysis was performed to assess the impact of the averaging on our findings, and results are included in the Supplementary Material S.3.

By smoothing the averaged daily activity, Fig. 1b shows the overall population mean PA intensity curve over 600 consecutive minutes, as well as the mean at each visit. As noted above, time “0” on the x-axis (Fig. 1b) indicates the common start time for all participants (after realignment), and 10 h of device wear are recorded as 0 to 600 min.

To account for the hierarchical structure of the data (visits within subjects) and its longitudinal nature in both predictors (PA) and health outcomes, we applied a longitudinal FPCA model to decompose densely sampled PA data, and a (functional) mixed effects regression model to explore the association between predictors and outcomes.

### 3.2 Longitudinal FPCA

Assuming no measurement error, a multilevel FPCA [12] can decompose an activity record $X_{ij}(t)$ for each subject i(i=1,2,…,N) at time t∈𝒟 (measured at the minute-level in the current analysis and 𝒟 can be treated as a set of grid points with length D) at each visit $j\left(j=1,2,\ldots ,n_{i}\right)$ in the form of

(1) $$X_{ij}\left(t\right)=\mu \left(t\right)+U_{i}\left(t\right)+V_{ij}\left(t\right),$$

where μ(t) represents the overall population mean function at $t.U_{i}(t)$ is the subject-specific deviation from the overall mean function. $V_{ij}(t)$ is the subject- and visit- specific deviation from the subject-mean function. The subject-specific variation can be further decomposed into the sum of a static part and a longitudinal part, which forms the basis of the longitudinal FPCA structure [17]. The detailed derivation of the model was given in Greven et al. and we will briefly describe it under our study setup. Specifically, for a two-level model, the functional input can be rewritten as,

(2) $$X_{ij}\left(t\right)=\mu \left(t\right)+U_{i0}\left(t\right)+U_{i1}\left(t\right)T_{ij}+V_{ij}\left(t\right),$$

where $U_{i0}(t)$ is the random functional intercept for subject $i,U_{i1}(t)$ is the random functional slope for subject i and $T_{ij}$ is the time at visit j for subject i, and in our application $T_{ij}$ has the form $T_{ij}=j$. To ensure the identifiability of the model, $U_{i}(t)=\left(U_{i0}(t),U_{i1}(t)\right)$ and $V_{ij}(t)$ are assumed to have mean zero and be mutually uncorrelated. $K_{U}(s,t)=\text{cov}\left\{U_{i}(s),U_{i}(t)\right\}$ and $K_{V}(s,t)=\text{cov}\left\{V_{ij}(s),V_{ij}(t)\right\}$ are covariance operators for the above random processes and $K_{U}$ and $K_{V}$ represent the corresponding covariance matrices for all s,t∈𝒟. Furthermore, for the subject-specific variation $K_{U}(s,t)$, the covariance operator between the bivariate process $U_{i}(t)$ has two parts: the auto-covariance $K_{U_{0}}(s,t),K_{U_{1}}(s,t)$ and the cross-covariance $K_{U_{01}}(s,t)$, which is represented as:

(3) $$K_{U}\left(s,t\right)=\left(\begin{array}{cc} K_{U_{0}}\left(s,t\right) & K_{U_{01}}\left(s,t\right)\\ K_{U_{01}}\left(t,s\right) & K_{U_{1}}\left(s,t\right) \end{array}\right).$$

Therefore, by Karhunen–Loéve expansion [19] on $U_{i}(t)$ and $V_{ij}(t)$, we obtain

(4) $$X_{ij}\left(t\right)=\mu \left(t\right)+\sum_{l=1}^{\infty }\left(1,T_{ij}\right)\xi_{il}\phi_{l}^{\left(1\right)}\left(t\right)+\overset{\infty }{\underset{m=1}{\sum }}\zeta_{ijm}\phi_{m}^{\left(2\right)}\left(t\right),$$

where $\phi_{l}^{\left(1\right)}(t)=\left(\phi_{l}^{U_{0}}(t),\phi_{l}^{U_{1}}(t)\right)\prime$ are the ordered eigenfunctions of $K_{U}(s,t)$ with corresponding eigenvalues $\lambda_{l}^{U}$ and $\phi_{m}^{\left(2\right)}(t)$ are the ordered eigenfunctions of $K_{V}(s,t)$ with corresponding eigenvalues $\lambda_{m}^{V}$. Specifically, eigenfunctions $\phi_{l}^{\left(1\right)}(t),l\in N$, are elements of $L^{2}[0,1]\times L^{2}[0,1]$ and satisfy the additive scalar product $\left\langle \left(f_{0},g_{0}\right),\left(f_{1},g_{1}\right)\right\rangle =\int_{0}^{1}\;f_{0}(t)g_{0}(t)\text{d}t+\int_{0}^{1}\;f_{1}(t)g_{1}(t)\text{d}t$. Details of the derivation can be found in Supplementary Material S.1. The corresponding principal component scores have the forms,

(5) $$\xi_{il}=\int \;U_{i0}\left(s\right)\phi_{l}^{U_{0}}\left(s\right)\text{d}s+\int \;U_{i1}\left(s\right)\phi_{l}^{U_{1}}\left(s\right)\text{d}s\text{ and }\zeta_{ijm}=\int \;V_{ij}\left(s\right)\phi_{m}^{\left(2\right)}\left(s\right)\text{d}s,$$

and are uncorrelated with mean zero and variances $\lambda_{l}$ and $\lambda_{m}$, respectively. In this way, the covariance operator of the longitudinal functional model becomes

(6) $$\begin{array}{cc} \text{Cov}\left\{X_{ij}\left(s\right),X_{ij\prime }\left(t\right)\right\} & =K_{U_{0}}\left(s,t\right)+\left(T_{ij}+T_{ij\prime }\right)K_{U_{01}}\left(s,t\right)+T_{ij}T_{ij\prime }K_{U_{1}}\left(s,t\right)+K_{V}\left(s,t\right)\delta_{jj\prime },\\ & \delta_{jj^{'}}=\{\begin{array}{cc} 1, & if\;j=j^{'}\\ 0, & \text{otherwise} \end{array}. \end{array}$$

Here, $\left\{K_{U_{0}}(s,t),K_{U_{1}}(s,t),K_{U_{01}}(s,t),K_{V}(s,t),s,t\in 𝒟\right\}$ are estimated by linearly regressing $X_{ij}(s)X_{ij^{'}}(t)$ on $\left(1,T_{ij},T_{ij^{'}},T_{ij}T_{ij^{'}},\delta_{jjj^{'}}\right)$ after mean-centering $X_{ij}(t)$. Eigen-functions and eigenvalues of the estimated covariance matrices $\left\{{\overset{ˆ}{K}}_{U_{0}},{\overset{ˆ}{K}}_{U_{1}},{\overset{ˆ}{K}}_{U_{01}},{\overset{ˆ}{K}}_{V}\right\}$ can be obtained via spectral decomposition, i.e., ${\overset{ˆ}{K}}_{U}=\sum_{l=1}^{2D}\;{\overset{ˆ}{\lambda }}_{l}^{U}{\overset{ˆ}{\phi }}_{l}^{\left(1\right)}{\left\{{\overset{ˆ}{\phi }}_{l}^{\left(1\right)}\right\}}^{'}$ and ${\overset{ˆ}{K}}_{V}=\sum_{m=1}^{D}\;{\overset{ˆ}{\lambda }}_{m}^{V}{\overset{ˆ}{\phi }}_{m}^{\left(2\right)}{\left\{{\overset{ˆ}{\phi }}_{m}^{\left(2\right)}\right\}}^{'}$. It is proved in Greven et al. [17] that if the time variable $T_{ij}$ is standardized to have zero mean and unit variance, i,e, $E\left(T_{ij}\right)=0$ and $\text{Var}\left(T_{ij}\right)=1$, the variation in $X_{ij}(t)$ can be decomposed additively and expressed with respect to the estimated eigenvalues, $\int_{𝒟}\;\text{var}\left(X_{ij}(t)\right)\text{d}t=\sum_{l=1}^{\infty }\;\lambda_{l}^{U}+\sum_{m=1}^{\infty }\;\lambda_{m}^{V}$. Usually a few most informative eigenfunctions are retained for further analysis. Criteria for selecting a finite number, $N_{U}$ and $N_{V}$, of subject- and visit-level eigenfunctions is discussed in Sect. 3.3. This finite sum then replaces the infinite sum in Eq. 4.

For fixed $N_{U}$ and $N_{V}$, Eq. 4 is a linear mixed model and we use the best linear unbiased prediction (BLUP) to obtain the predicted principal component scores $\xi_{il}$ and $\zeta_{ijm}$. Let $\overset{ˆ}{\beta }=\left({\overset{ˆ}{\xi }}_{11},\ldots ,{\overset{ˆ}{\xi }}_{1N_{U}},\ldots ,{\overset{ˆ}{\xi }}_{N1},\ldots ,{\overset{ˆ}{\xi }}_{NN_{U}},{\overset{ˆ}{\zeta }}_{111},\ldots ,{\overset{ˆ}{\zeta }}_{11N_{V}},\ldots ,{\overset{ˆ}{\zeta }}_{Nn_{N}1},\ldots ,{\overset{ˆ}{\zeta }}_{Nn_{N}N_{V}}\right)$, then estimated BLUP of $\overset{ˆ}{\beta }$ is given by,

(7) $$\hat{\beta }=\left(Z\prime Z\right)-1Z\prime X,$$

where $Z=\left[E_{I}⨂\Phi^{U_{0}}+T⨂\Phi^{U_{1}}|I⨂\Phi^{V}\right],\;E_{I}={\left(\delta_{ijh}\right)}_{ij=11,\ldots ,Nn_{N}};h=1,\ldots ,N,\;T={\left(T_{ij}\delta_{ijh}\right)}_{ij=11,\ldots ,Nn_{N}};h=1,\ldots ,N,\;\Phi^{U_{0}}={\left\{\phi_{l}^{U_{0}}(t)\right\}}_{t\in 𝒟,l=1,\ldots ,N_{U}},\;\Phi^{U_{1}}={\left\{\phi_{l}^{U_{1}}(t)\right\}}_{t\in 𝒟,l=1,\ldots ,N_{U}}$, $\;\Phi^{V}={\left\{\phi_{l}^{V}\left(t\right)\right\}}_{t\in 𝒟,l=1,\ldots ,N_{U}}$, I is the $\sum_{i}\;N_{i}$ dimensional diagonal matrix with element 1, $X=\left[{\left\{X_{11}(t)\right\}}_{t\in 𝒟},\ldots ,{\left\{X_{1N_{1}}(t)\right\}}_{t\in 𝒟},\ldots ,{\left\{X_{N1}(t)\right\}}_{t\in 𝒟},\ldots ,{\left\{X_{Nn_{N}}(t)\right\}}_{t\in 𝒟}\right],\;$ and ⊗ denotes the Kronecker product of matrices. ${\left(\delta_{ijh}\right)}_{ij=11,\ldots ,Nn_{N}};h=1,\ldots ,N$ denotes the indicator matrix with entries $\delta_{ijh}$ at row $ij,i=1,\ldots ,N,j=1,\ldots ,n_{i}$ and column h,h=1,…,N, with $\delta_{ijh}=1$ if i=h and $\delta_{ijh}=0$ otherwise.

Although the methods were described in detail in Greven et al. [17], our no measurement error setting differs slightly from the model specified in the original paper. Therefore, for completeness we provide the proof of the BLUP derivation (see Supplementary Material S.1). In addition, we implemented simulation studies, in order to illustrate the applicability of the proposed methods, and to evaluate how higher values for subject-level versus visit-level variation (and vice-versa) influenced goodness of fit of the various model components. The simulation assumptions and results can be found in Supplementary Material S.2. We discuss a few key results here. The boxplots of the estimated normalized errors of principal component scores show all parameters are unbiasedly estimated, demonstrating agreement with the simulation results in Greven et al. [17].

In addition to results from parameter estimation, we include residual mean square error (MSE) results in Supplementary Material S.2 from each of the two simulation scenarios with three ways of computing residuals $R_{ij}(t)$, the residuals from subject-level $X_{ij}(t)-U_{i}(t)$, the residuals from visit-level $X_{ij}(t)-V_{ij}(t)$ and the overall residuals $X_{ij}(t)-U_{i}(t)-V_{ij}(t)$. Let M be the total number of observations, the residual MSE for one simulation replicate is defined as $\frac{1}{M}\sum_{i,j}\;{\left(\sum_{t}\;\left|R_{ij}(t)\right|\right)}^{2}$, which in fact reflects the total mean squared count difference per observation between the predicted and observed activity curves, when using only level 1 predictions, only level 2 predictions or both. Thus this mean-squared error represents the goodness-of-fit of the model when using different fitted components. Since larger eigenvalues indicate more explained variability, the goodness of fit of the subject- versus visit-level predictions depends on which component has the largest eigenvalue, as seen from the two simulation scenarios.

The simulations confirm that the estimated principal component scores $\xi_{il},\;\zeta_{ijm}$ and hence the decomposed random processes $U_{i}(t),V_{ij}(t)$ obtained from the longitudinal FPCA model are reasonably accurate at recapitulating the observed temporal patterns of subject- and visit-level PA. We will use the PA patterns as predictors of outcomes in regression models, as detailed in the next section.

For data observed with white noise, denoted as ${\tilde{X}}_{ij}(t)=X_{ij}(t)+\epsilon_{ij}(t)$, as suggested in Shou et al. [29], smoothing the raw data ${\tilde{X}}_{ij}(t)$ can be implemented before performing the longitudinal FPCA. Since the main purpose of this study is to explore the associations between general activity patterns and health outcomes, smoothing the raw inputs is preferable for these densely sampled accelerometer inputs. We implemented a thin plate regression spline smoother to the original data with 10 basis functions. As sensitivity analysis we also evaluated the impact of under- or over-smoothing by varying the number of basis vectors.

### 3.3 Regression Model

With results from the longitudinal FPCA, the associations between physical activity and health outcomes are explored via regression modeling. Two regression models, regression modeling with principal component scores (PCR) and functional regression model with decomposed random processes (fPCR), are implemented in our analysis, and briefly discussed in this section. The first regression model directly incorporates subject- and visit-level principal component scores as predictors. To account for the repeated measures pattern in outcomes $Y_{ij}$, we use linear mixed models. Thus, the PCR is given as,

(8) $$E\left(Y_{ij}\right)=\alpha_{0}+\alpha_{1}I(j>1)+b_{i}+\overset{N_{U}}{\underset{l}{\sum }}\beta_{l}^{U}\xi_{il}+\overset{N_{V}}{\underset{m}{\sum }}\beta_{m}^{V}\zeta_{ijm}+\text{ other covariates ,}$$

where the α and β parameters are fixed effects, namely, $\alpha_{0}$ is the intercept at baseline visit and $\alpha_{1}$ is the mean change at follow-up visits, and βs quantify associations between diurnal activity pattern (captured via subject- and subject-visit principal components and scores); $b_{i}$ is a subject-specific random effect and the assumptions $b_{i}\sim N\left(0,\epsilon^{2}\right)$ and $b_{i}$ is conditionally independent of $Y_{ij}$ hold. ‘other covariates’ refers to covariates which one might adjust for, which will depend on the particular study. In our application to the MENU study, we adjusted for age, ethnicity, smoking status, and follow-up visit. The number of components $N_{U}$ and $N_{V}$ are chosen to explain a pre-specified proportion of variance and in our application, we will choose enough components to explain over 85% variance. The fixed effects $\beta^{U},\beta^{V}$ and random effects $b_{i}$ are estimated with R package lme4 [5].

Another regression model we consider in this paper is the fPCR, which replaces principal component scores with functional curves as predictors. The functional predictors include between-subject variation $U_{i}(t)$ and between-visit variation $V_{ij}(t)$, which can be reconstructed in the form of $U_{i}(t)=\sum_{l}^{N_{U}}\;\xi_{il}\phi_{l}^{\left(1\right)}(t)$ and $V_{ij}(t)=\sum_{m}^{N_{V}}\;\zeta_{ijm}\phi_{m}^{\left(2\right)}(t)$. Here $U_{i}(t)$ is interpreted as the overall trend for subject i while subject-visit variation is captured by $V_{ij}(t)$. The fPCR model then has the form,

(9) $$E\left(Y_{ij}\right)=\alpha_{0}+\alpha_{1}I(j>1)+b_{i}+\int \beta_{U}\left(t\right)U_{i}\left(t\right)\text{d}t+\int \beta_{V}\left(t\right)V_{ij}\left(t\right)\text{d}t+\text{other covariates}.$$

The αs and $b_{i}$ have similar interpretation as the PCR model. The β parameters are now represented as smooth coefficient functions $\beta_{U}(t)$ and $\beta_{V}(t)$, and are estimated using penalized spline methods in our application via the R package mg cv [33, 34].

The estimated principal component scores quantify the extent to which a subject or subject-visit subscribe to the corresponding temporal patterns delineated by the principal components. Thus, as noted in Gertheiss et al. [15], by incorporating principal component scores as covariates, the PCR assesses associations between activity patterns and outcomes, and thus may have intuitive appeal. However, PCR is subject to overfitting, due to the need to a priori choose the number of principal components $\left(N_{U}\right.$ and $\left.N_{V}\right)$. The fPCR, on the other hand, is more flexible and can yield a more nuanced interpretation, especially when the coefficient functions are significant for some time domains. We will demonstrate the comparison in later sections.

## 4 Results

### 4.1 Sample Characteristics

The study population had average age of 50.8 years (SD 9.9), with range 22–72 years; 81.6% were non-Hispanic and 69% had no history of smoking. In addition, summary information of insulin, C-reactive protein (CRP) and body mass index (BMI) across the three visits are listed in Table 1. All three outcomes present a decreasing trend after the baseline visit, indicating improved health status at follow-up.

Summary statistics of physical activity by visit are included in Table 1 as well. Total magnitude computes the averaged sum of activity counts for a participant at each visit and is a measure of total activity. We also present standard metrics for PA study, including daily sedentary time and MVPA time. The increasing average movement magnitudes, shorter sedentary time and longer MVPA at follow-up visits imply that on average, participants increased physical activity after enrolling in this study. Boxplots of daily average activity magnitudes at individual level are provided in Supplementary Material S.5, which further establish an increase in PA magnitudes after baseline visits. Meanwhile, no notable seasonal variability is detected for this one-year longitudinal study, which is unsurprising for a study conducted in southern California.

### 4.2 Functional Physical Activity Patterns

#### 4.2.1 Extracting Functional Principal Components

For functional PA inputs, we fitted the longitudinal FPCA on averaged daily activity magnitudes, given the longitudinal design of our study. The number of principal components for subject (level 1) and visit (level 2) level patterns, i.e., $N_{U}$ and $N_{V}$, were chosen based on the percentage of explained variation, and an attempt to achieve balance between under-fitting of the covariance matrix and over-fitting the regression model. In this study, we retained sufficient components to ensure that 95% overall variation in activity patterns could be explained.

#### 4.2.2 Variance Explained by Level 1 and Level 2 Principal Components

Five level 1 principal components and nine level 2 principal components were retained to explain 95% of activity variation. The detailed results are included in Supplementary Material S.5, which gives cumulative variation explained for the first five components at each level. For the level 1 principal components, the first component for subject-specific process U explains 25% of the variation. Also, within U, most of the variation is explained by the random functional intercept $U_{0}$ (38.55%) while the random functional slope only explains < 5% of the variation, suggesting that variation between subjects is largely captured by overall PA amount rather than by longitudinal trends. Another 25% of the variation is explained by the first principal component of the level 2 visit-specific process V. Overall, the first five components of the subject-level process and visit-level process each explain around 43% variation, indicating that they capture equal amount of variation in the data.

#### 4.2.3 Interpreting Level 1 and Level 2 Principal Components

**Fig. 2:**
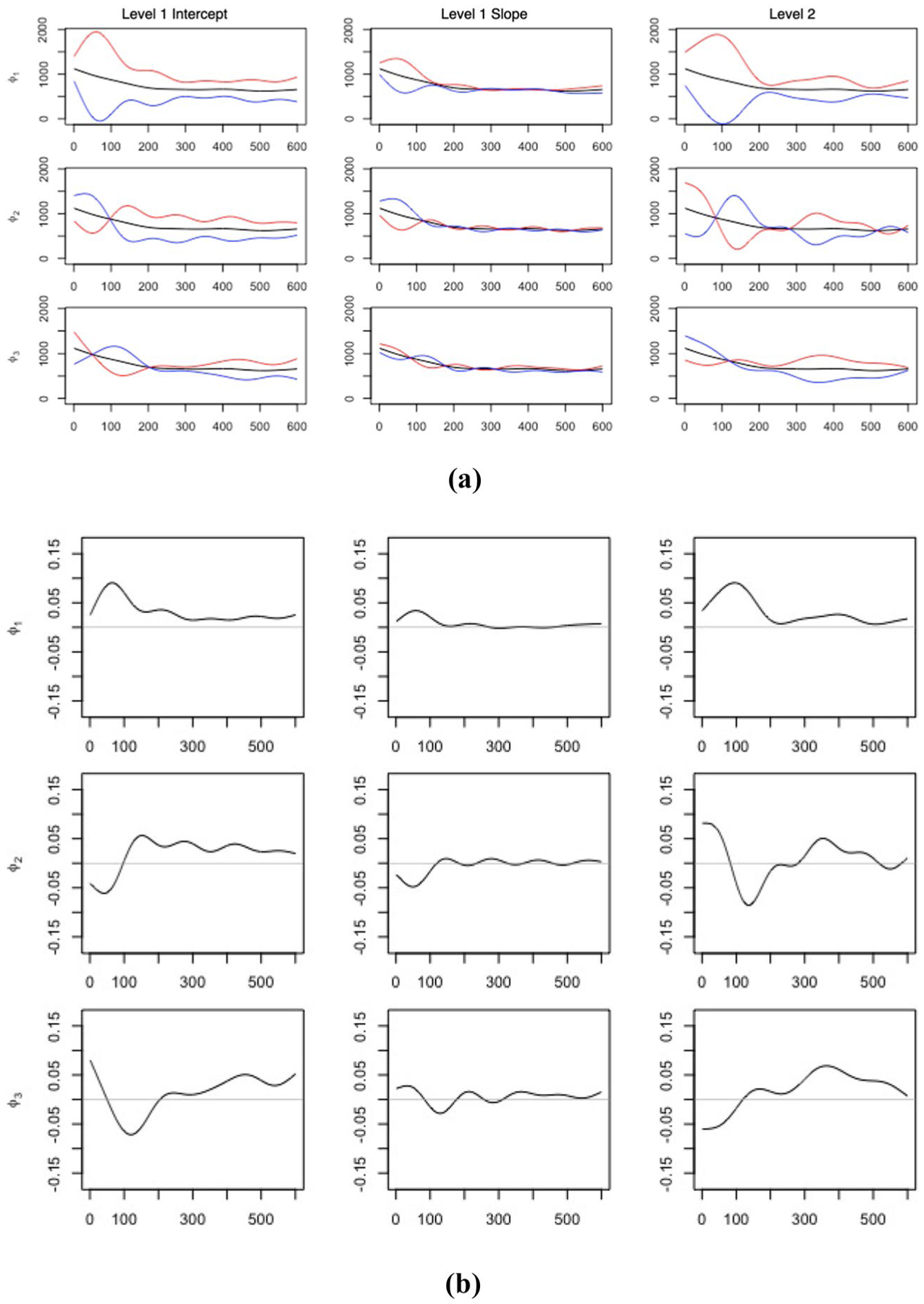
The first three estimated principal components for the random intercept (left column), random slope (middle column) and visit-specific process (right column). The plots give the **a** overall mean value curve μ(t) (black) with addition (red) or subtraction (blue) of 2 square root of eigen values multiplying first or second level principal component curves $\left(\pm 2\sqrt{\lambda_{l}}\phi_{l}^{U}\right.$ or $\left.2\sqrt{\lambda_{m}}\phi_{m}^{V}\right)$ respectively; **b** estimated eigenfunctions of the first three principal components. The horizontal gray line represents 0

Figure 2 illustrates the first three estimated principal components for the random intercept, random slope and visit-specific process by columns. The plots in Fig. 2a depict the overall mean curve μ(t) (black curve) with adding (red) or subtracting (blue) the value of 2 square root of eigen values multiplying first (or second level) principal component curves (i.e., $\pm 2\sqrt{\lambda_{l}}\phi_{l}^{U}$ or $2\sqrt{\lambda_{m}}\phi_{m}^{V}$), respectively. The plots in Fig. 2b represent the eigenfunctions themselves and together these sets of plots can be used to interpret the PA patterns associated with each principal component. For instance, the first level 1 intercept principal component (top left in Fig. 2b) is above the horizontal line at 0 throughout the 600 min, and represents an overall vertical shift of the mean activity curve. As seen in the corresponding top left plot in Fig. 2a, the red curve, which represents adding (a multiple of) this principal component to the mean, is always higher than the mean curve. Thus a high score on this component indicates that a participant is on average more physically active throughout the time interval compared to one with a lower value. It is also observed that the peak of this curve appears at around an hour after wearing, showing that early activity is more notable for capturing between subject variability. The first level 1 random slope process curve (top middle plot of Fig. 2a and b) show a similar pattern but with smaller variance. We also note that in the Karhunen–Loéve expansion, the level 1 intercept and slope eigenfunctions share the same level 1 score. This implies that a subject with a higher score in the first level 1 component will not only be more active overall, but also show a higher increase across visits.

The other level 1 components illustrate variation in timing of activity and identify periods of higher versus lower activity. For instance, the second level 1 intercept component (middle left plot in Fig. 2b) is negative (i.e., below the horizontal line at 0) for the first 100 min and then becomes positive for the remaining 500 min, which indicates a contrast between earlier versus later activity. This is further evident in the middle left panel in Fig. 2a, where the red curve is below the mean for the first 100 min and then switches to being above the mean. A high positive score on this component would signify less activity in the early period (i.e., first 100 min) with increased activity later on.

The first level 2 visit-specific curve, on the other hand, captures visit-to-visit shift from the subject-level curve. A participant with a higher score on this component would be more physically active longitudinally, based on the red curve in the top right panel of Fig. 2a being always above the mean, or equivalently the curve in Fig. 2b being always positive (i.e., above the horizontal line at 0). The peak for this curve appears at around 100 min and shows a delayed pattern compared with the first level 1 process, suggesting that visit-to-visit variation is more pronounced at the later morning time.

#### 4.2.4 Illustrative Examples of Principal Component Scores

**Fig. 3:**
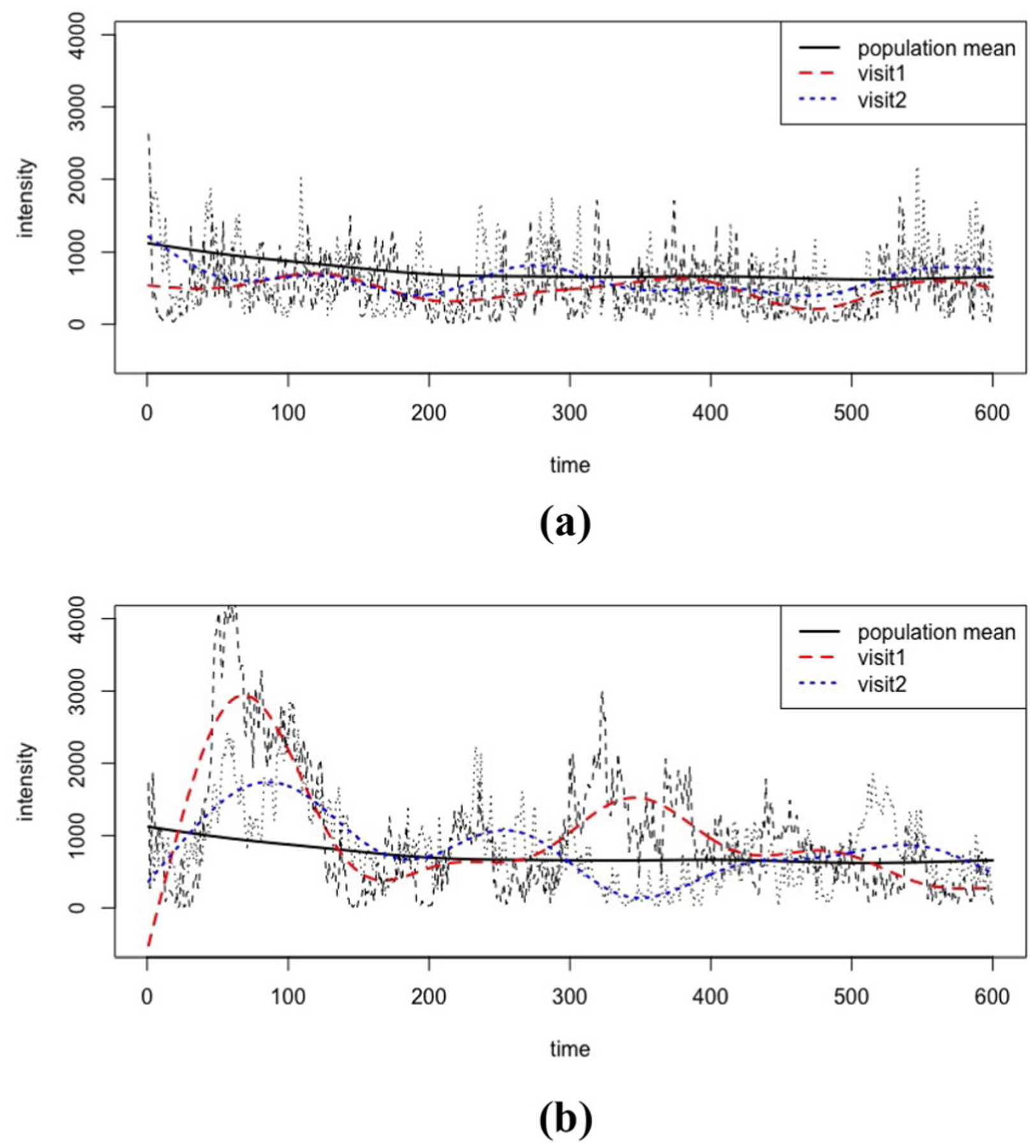
Two examples of PA records with raw count inputs (black) and estimated curves at each visit (red, blue): **a** is an example with a large first level 1 principal component score but a small first level 2 principal component score; **b** is an example with a small first level 1 principal component score but a large first level 2 principal component score

For each principal component, the corresponding principal component score quantifies the magnitude of the temporal pattern associated with that component. Thus, the principal component score itself can be used as a quantified indicator of the variation in PA records. To demonstrate this, two examples are given in Fig. 3. In Fig. 3a, an individual example with a large first level 1 principal component score but a small first level 2 principal component score is given, showing a significant early-time bounce at both visits, with little variation between visits. Figure 3b presents an individual example with a small first level 1 principal component score but a large first level 2 principal component score and in this case, the large variation between visits is apparent. Detailed decomposition figures are included in Supplementary Material S.4, illustrating a step-wise reconstruction after decomposition. These two examples to some extent also reflect our simulation results of residual MSEs (Sect. 3.2, Supplementary Material S.2), since larger eigenvalues are more likely to have higher scores. Both examples further illustrate Fig. 2, and demonstrate how level 1 versus level 2 principal component scores are useful for evaluating between- and within-subject activity patterns. It is also evident that the fitted (smoothed) FPCA curves track the original activity counts reasonably well, indicating that our fitted model provides a good fit to the data.

### 4.3 Regression Patterns: Associations Between Physical Activity and Health Outcomes

#### 4.3.1 Principal Component Regression (PCR) Using Principal Component Scores

In regression analysis, we first implemented the PCR models to explore the association between PA and health outcomes. In these models, physical activity patterns are modeled with estimated principal component scores as predictors, similar to the model in Eq. 8. Table 2 gives the results of the regression model, adjusting for baseline age, ethnicity, smoking status, and a logical variable indicating whether the participant is at a follow-up visit. The model accounts for individual variation by adding a random intercept $b_{i}$. The regression coefficients of the visit indicator and the first two principal component scores for both levels, which explained over 70% of variance jointly, are given in Table 2. For the purpose of comparing and interpreting model coefficients, all level 1 and level 2 principal component scores are also scaled to be in the range of 0 and 1.

All three health outcomes are negatively associated with the visit indicator, reflecting decreasing levels at follow-up, i.e., after the intervention. The first level 1 principal component scores are negatively associated with insulin, CRP and BMI, suggesting that more PA is associated with lower levels of these health outcomes, i.e., higher PA is associated with better metabolic health. In addition, the first level 2 principal component scores are negatively associated with insulin and CRP, suggesting that increased PA between visits (within an individual) is associated with greater decline in biomarkers.

To compare PCR to standard methods which use physical activity summaries, we also fitted a mixed effect regression model by including total (averaged) activity counts and MVPA as predictors respectively (Supplementary Material S.5), whose values were also scaled to be in the range of 0 and 1. It shows that both total activity counts and MVPA also exhibit a negative association with health outcomes, which supports findings from PCR models. However, the analysis based on daily summary PA estimates such as total activity counts or MVPA fails to capture the temporal aspect of PA accumulation, e.g., the level 1 first principal component of the intercept process suggests that peak activity occurred at around an hour from the start time. We further elucidate on these and other differences between standard and functional regressions methods in Sect. 5.

#### 4.3.2 Functional Principal Component Regression (fPCR)

**Fig. 4:**
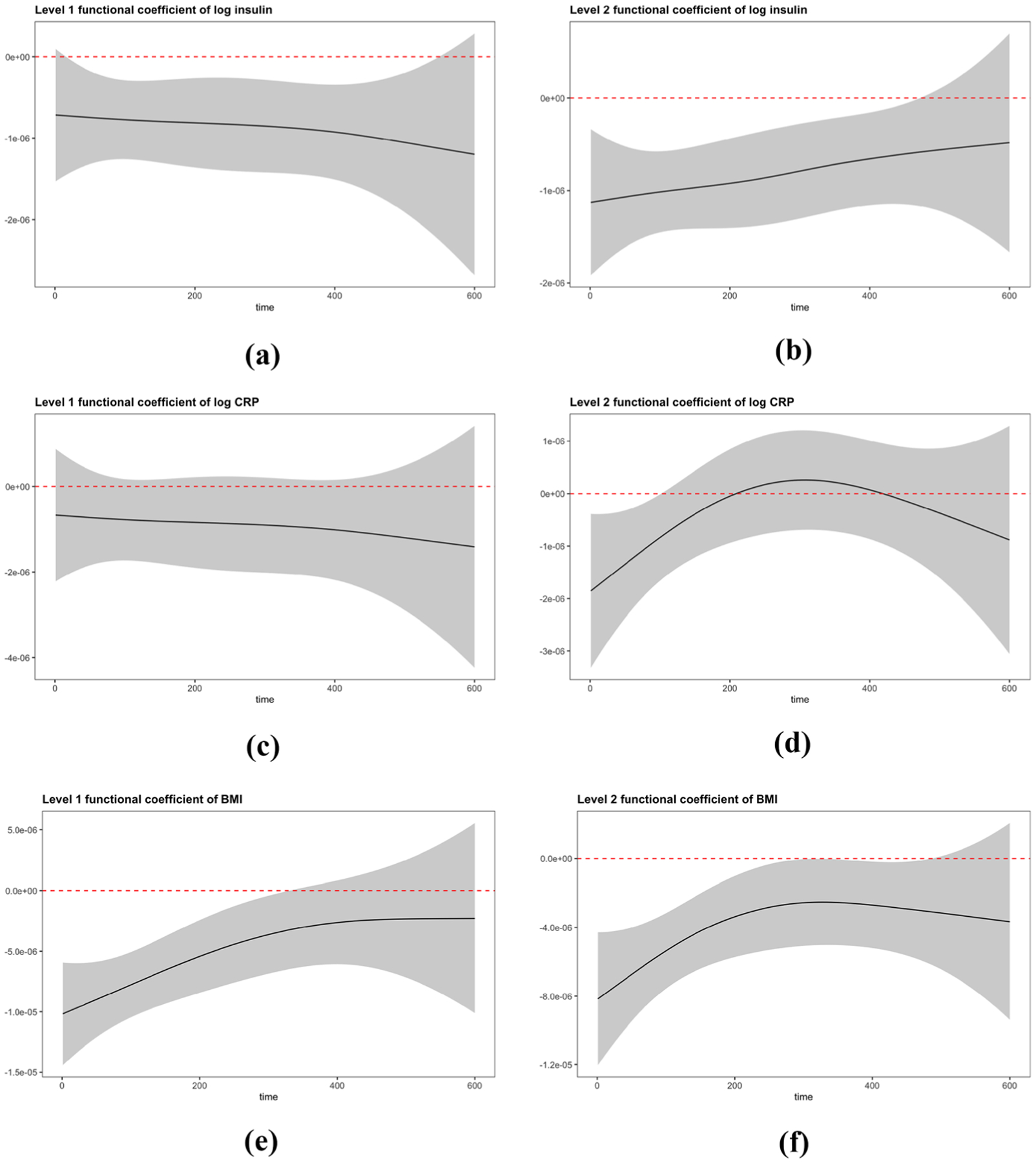
Estimated functional coefficients curve (with 95% pointwise confidence intervals) when functional principal component regression (fPCR) models with functional predictors $U_{i}(t),V_{ij}(t)$ and random intercept $b_{i}$ are fitted (adjusted for baseline age, ethnicity, smoking history and visit indicator)

Along with the PCR, fPCR models were also implemented to better exemplify the diurnal association between PA and health outcomes. Figure 4 presents the estimated coefficient functions with 95% pointwise confidence intervals. The coefficients at a given time-point (on the x-axis) are considered significant if the 95% confidence limits at that time do not cross the reference horizontal line at y=0. As shown in the figure, the coefficient functions for the level 1 and 2 processes for log(insulin) are negative and significant for most time-points of the day, suggesting that participants with more PA (irrespective of time of accumulation) than the “average participant” or the “previous visit” tended to have lower insulin. These effects are stronger (and significant) if PA occurred during earlier times (of day) for the level 2 coefficients for log(insulin), indicating stronger effects for visit-to-visit change in PA earlier in the day. Similar results are observed for BMI, although the level 1 coefficients are minute-wise significant up to approximately the first 300 min (see x-axis), whereas the level 2 coefficients are significant throughout the day. Interestingly, both level 1 and level 2 coefficients for BMI show an initial increasing pattern with leveling off later, suggesting that PA earlier (rather than later) in the day is more beneficial for reducing weight. The effect of PA on log(CRP) is not significant in the first level but shows a pointwise negative association in the second level coefficients only during the first 100 min of wear.

## 5 Discussion

In this work we have demonstrated the use of functional principal component analysis to extract patterns of physical activity from accelerometer data, and use these patterns to evaluate associations between PA and health outcomes. Functional data analysis provides a rich statistical framework for modeling the variation of physical activity curves. While summary statistics such as weekly total activity counts or MVPA provide aggregated metrics, functional data analysis can unravel temporal patterns, and presents varying activity patterns of individuals throughout the day.

Conventional approaches usually summarize statistical characteristics from accelerator data (e.g., mean weekly MVPA), and then use these summaries to examine longitudinal associations between PA and health outcomes. These methods ignore the full spectrum of activity magnitude trajectories. On the other hand, functional modeling allows a more robust decomposition of the original accelerometer inputs, and thus could provide a richer framework for examining PA-health associations. From mixed effect regression models by including conventional summary measures of PA as predictors, such as total activity counts and MVPA, the results (Supplementary Material S.5) show high concordance with coefficients of the first level 1 and level 2 principal components scores from the PCR models (Table 2), which in fact can be interpreted as measuring the average amount and visit-visit change of activity for each individual. However, the total activity counts and MVPA in the model did not explicitly separate the subject-level (level 1) and between-visit variations (level 2).

Importantly, summary PA measures such as weekly total activity or MVPA cannot identify associations between diurnal variation in PA accumulation and health, which is exemplified by fPCR to further extend the regression model, using smooth coefficient functions to explain predictors’ influence on health outcomes. From the coefficient functions for the health outcomes (Fig. 4), the fPCR shows advantages by providing a trend of changing coefficients over time (of day). The level 1 and level 2 functional coefficients are negatively associated with the outcomes, which is in conformity with the findings for both, standard summary measures and PCR. However, from fPCR we are also able to discern that the level 1 and 2 coefficients for BMI, albeit negative, increase during the day, indicating that earlier activity is potentially more beneficial for weight management among overweight women. Interestingly, while also negative, the level 1 coefficient function for log(insulin) is relatively stable throughout the day, suggesting that PA, irrespective of time of accumulation, is equally beneficial for controlling insulin level. Thus, the timing of activity during the day may differentially impact biomarker outcomes, a fact that would be useful for designing personalized activity interventions.

In addition, the implementation of longitudinal models emphasizes the statistical analysis of cross-visit variation in both functional PA predictors and scalar health outcomes. On one hand, longitudinal FPCA reveals how different PA patterns within one participant reflect either a more active or sedentary style, as the examples shown in Fig. 3. On the other hand, the application of the fPCR, extends the interpretation of the regression coefficients to minute-level at each visit. This is advantageous because the PA inputs and predicted coefficients are correspondingly matched in the same scale. In contrast with ordinary regression coefficients of principal component scores (Table 2), which provide the association between the full daily activity profile and health outcomes, the fPCR approach treated regression coefficients as smooth functions of time and computed an estimated coefficient at each time-point. As it was mentioned in Dziak et al. [14], a motivation of incorporating coefficient curves is to look for a period of time during which the predictors are more strongly associated with outcomes. However, we urge caution when interpreting time intervals, as our results are based on pointwise 95% intervals, and thus could be subject to increased Type 1 error when considering multiple time-points.

Another advantage of using fPCR compared with PCR is consideration of the number of functional principal components. In PCR, the number of principal components to retain is usually determined based on explaining sufficient variability in functional inputs, which might result in overfitting the regression model. On the contrary, when fitting a fPCR model, this is not an important concern, since a penalty is used to avoid overfitting. Construction of random process predictors often requires a large enough number of principal components to capture important features, which makes the fPCR more robust when the first few principal components do not explain enough variation in the predictor. We also note that our fPCR results are generally robust to varying the smoothing parameters. The results are essentially unchanged (data not shown) when we under smoothed, i.e., increased the number of basis vectors from 10 to 15 or 20. On the other hand, over-smoothing (e.g., 5 basis functions) results in attenuated effects, possibly due to a loss of information.

Further research is needed to address several limitations of this work. Firstly, we only implemented the model on visit-level data averaged across days. Additional methodological work, beyond the scope of the current investigation, is required to extend our current model to a three-level longitudinal FPCA model, which can be fitted with daily inputs. For our application, a sensitivity analysis (Supplementary Material S.3) demonstrated that averaging the day-level PA inputs did not materially affect our analysis or results. Also, we realigned all PA profiles to a common start time. We believe that this realignment initializes each record at the participant’s own starting time which seems more appropriate for capturing an individual participant’s wake-time activity patterns, compared to using an arbitrary and fixed clock time for all individuals. Even so, more advanced analytic and registration approaches may need to be considered, especially when performing analysis directly on the day-level data, and particularly for applications where the variation could be larger within this level. Thirdly, we used pointwise 95% confidence intervals, which are specific to a given time-point. Estimating confidence bands for functional data that will account for all time-points simultaneously, is an area of active research; we will consider these extensions in future work. Also, although our findings could be used to optimally design timing of PA interventions, we recommend replication in independent cohorts, given the exploratory nature of principal components analysis. Finally, the functional approach does not specifically delineate levels of activity intensity, e.g., MVPA from light activity. Compositional data analysis, an emerging and highly relevant area of research [13], allows evaluation of different (correlated) activities (e.g., sleep, sedentary time, MVPA) in the same model. While the focus of our functional approach is to elicit diurnal patterns of overall activity, it may be interesting to incorporate multiple behaviors into a functional model, thus leveraging the strengths of both functional and compositional data analysis methodologies. We leave this to future investigations.

## 6 Conclusion

In summary, our longitudinal FPCA model offers a new approach for analyzing the association of physical activity patterns with health outcomes. We have demonstrated that functional modeling can not only yield comparable results with traditional PA summary statistics with longitudinal outcomes, but also provide further information on the time domain of daily activities, including the association between PA effects at certain times of the day and health outcomes. These findings could be useful for providing individualized activity guidelines for overweight women and to promote health and weight control. Importantly, the use of wearable sensors for PA is becoming more and more common in public health research. Use of functional data methods to explore PA patterns could offer a useful complement to summary-based PA measures.

## Supplementary Material

Supplementary material

## Figures and Tables

**Table 1:** Summary statistics of health outcomes and daily physical activity at each visit (mean (SD))

	Overall	Baseline	6 months	12 months
*Health outcomes*				
Insulin (pg/mL)	13.44 (7.68)	14.49 (8.26)	12.63 (6.60)	12.98 (7.90)
CRP (|ig/mL)	3.99 (4.70)	4.86 (5.28)	3.64 (3.88)	3.28 (4.60)
BMI	31.79 (4.12)	33.50 (3.34)	30.91 (3.98)	30.74 (4.43)
*Activity magnitude*				
Total magnitude (x10^5^)	4.38 (2.02)	4.04 (1.75)	4.63 (2.18)	4.55 (2.11)
Sedentary time (min)	312.33 (87.81)	318.22 (87.62)	308.93 (86.43)	308.14 (89.17)
MVPA time (min)	40.62 (32.42)	34.43 (27.07)	45.42 (35.62)	43.61 (33.92)

**Table 2:** Linear mixed effect regression results of health outcomes on the first two-level 1 and level 2 principal component scores of physical activity

Outcome	Predictor	Coefficient estimate	SE	Confidence interval
Log (insulin)	PC11	− 0.15	0.04	(− 0.23, − 0.06)
	PC12	− 0.08	0.03	(− 0.14, − 0.03)
	PC21	−0.16	0.04	(− 0.24, − 0.08)
	PC22	0.03	0.03	(− 0.03, 0.08)
	visit > 1	− 0.09	0.03	(− 015, − 0.04)
Log(CRP)	PC11	− 0.23	0.09	(− 0.41, − 0.06)
	PC12	− 0.11	0.06	(− 0.24, 0.01)
	PC21	− 0.09	0.08	(− 0.24, − 0.06)
	PC22	− 0.04	0.05	(− 0.14, 0.06)
	visit > 1	− 0.36	0.05	(− 0.45, − 0.27)
BMI	PC11	− 0.74	0.29	(− 1.31, − 0.17)
	PC12	− 0.31	0.23	(− 0.75, 0.14)
	PC21	− 0.23	0.21	(− 0.66, 0.18)
	PC22	− 0.02	0.14	(− 0.3, 0.25)
	Visit > 1	− 2.56	0.13	(− 2.81, − 2.3)

* Adjusted for baseline age, ethnicity, smoking history and visit indicator.

** PC11 represents the first level 1, PC12 the second level 1, PC21 the first level 2, and PC22 the second level 2 principal component scores
