# Supplementary material for: Longitudinal Associations Between Timing of Physical Activity Accumulation and Health: Application of Functional Data Methods
Source: Stat Biosci. Author manuscript; Available in PMC 2024 Jul 1. (PMC10299778; doi:10.1007/s12561-022-09359-1)
Supplement: Supplementary material [file NIHMS1905764-supplement-Supplementary_material.pdf]

Supplementary Material for "Longitudinal associations between timing of physical activity accumulation and health: Application of functional data methods" by

Wenyi Lin[1], Jingjing Zou[1], Chongzhi Di[2], Dorothy D. Sears[3,4,5], Cheryl L Rock[1] and Loki Natarajan[1*]

[1*]Division of Biostatistics, Herbert Wertheim School of Public Health and Longevity Science, University of California, San Diego, CA, USA.

[2]Public Health Sciences Division, Fred Hutchinson Cancer Research Center, Seattle, WA, USA.

[3]College of Health Solutions, Arizona State University, Phoenix, AZ, USA.

[4]Department of Medicine, University of California, San Diego, CA, USA.

[5]Department of Family Medicine, University of California, San Diego, CA, USA.

*Corresponding author(s). E-mail(s): lnatarajan@health.ucsd.edu;

S.1 Additional Proof

- If $K_U(s,t) = \begin{pmatrix} K_{U_0}(s,t) & K_{U_{01}}(s,t) \\ K_{U_{01}}(t,s) & K_{U_1}(s,t) \end{pmatrix} = \sum_{l=1}^{\infty} \lambda_l^U \phi_l^{(1)}(s)\phi_l^{(1)}(t)'$, where $\phi_l^{(1)}(t) = (\phi_l^{U_0}(t), \phi_l^{U_1}(t))'$, then $K_{U_0}(s,t) = \sum_{l=1}^{\infty} \lambda_l^U \phi_l^{U_0}(t)\phi_l^{U_0}(s)$, $K_{U_1}(s,t) = \sum_{l=1}^{\infty} \lambda_l^U \phi_l^{U_1}(t)\phi_l^{U_1}(s)$ and $K_{U_{01}}(s,t) = \sum_{l=1}^{\infty} \lambda_l^U \phi_l^{U_0}(t)\phi_l^{U_1}(s)$.
  Proof:

$$K_U(s,t) = \sum_{l=1}^{\infty} \lambda_l^U \phi_l^{(1)}(s)\phi_l^{(1)}(t)'$$

$$= \sum_{l=1}^{\infty} \lambda_l^U (\phi_l^{U_0}(s), \phi_l^{U_1}(s))(\phi_l^{U_0}(t), \phi_l^{U_1}(t))'$$
$$= \sum_{l=1}^{\infty} \lambda_l^U \begin{pmatrix} \phi_l^{U_0}(s)\phi_l^{U_0}(t) & \phi_l^{U_0}(s)\phi_l^{U_1}(t) \\ \phi_l^{U_0}(t)\phi_l^{U_1}(s) & \phi_l^{U_1}(s)\phi_l^{U_0}(t) \end{pmatrix}$$
$$= \sum_{l=1}^{\infty} \begin{pmatrix} \lambda_l^U \phi_l^{U_0}(s)\phi_l^{U_0}(t) & \lambda_l^U \phi_l^{U_0}(s)\phi_l^{U_1}(t) \\ \lambda_l^U \phi_l^{U_0}(t)\phi_l^{U_1}(s) & \lambda_l^U \phi_l^{U_1}(s)\phi_l^{U_0}(t) \end{pmatrix}$$
$$= \begin{pmatrix} K_{U_0}(s,t) & K_{U_{01}}(s,t) \\ K_{U_{01}}(t,s) & K_{U_1}(s,t) \end{pmatrix}.$$

- **Proof of Equation 7**. For given eigenfunctions, eigenvalues, the BLUP for principal component scores $\hat{\boldsymbol{\beta}} = (\hat{\xi}_{11}, \ldots, \hat{\xi}_{1N_U}, \ldots, \hat{\xi}_{N1}, \ldots, \hat{\xi}_{NN_U}, \hat{\zeta}_{111}, \ldots, \hat{\zeta}_{1J_11}, \ldots, \hat{\zeta}_{N1N_V}, \ldots, \hat{\zeta}_{Nn_NN_V})$ has a usual form as,

$$\hat{\boldsymbol{\beta}} = \boldsymbol{\Lambda Z}'(\boldsymbol{Z\Lambda Z}')^{-1}\boldsymbol{X},$$

where $\boldsymbol{\Lambda} = blockdiag\{\boldsymbol{I}_N \bigotimes diag(\lambda_1^U, \ldots, \lambda_{N_U}^U), \boldsymbol{I}_n \bigotimes diag(\lambda_1^V, \ldots, \lambda_{N_V}^V)\}$, $n = \sum_{i=1}^N n_i$. When $D > N_U + n_i * N_V$, $\boldsymbol{Z\Lambda Z}'$ is not invertible and only the generalized inverse of $\boldsymbol{Z\Lambda Z}'$ can be used[2]. For our implementation, N_U and N_V are in general small numbers and the length of grid points of time is always significantly larger. In this case, $\hat{\boldsymbol{\beta}} = \boldsymbol{\Lambda Z}'(\boldsymbol{Z\Lambda Z}')^{-1}\boldsymbol{X} = \boldsymbol{\Lambda}^{1/2}(\boldsymbol{\Lambda}^{1/2}\boldsymbol{Z}'\boldsymbol{Z}\boldsymbol{\Lambda}^{1/2})^{-1}\boldsymbol{\Lambda}^{1/2}\boldsymbol{Z}'\boldsymbol{X} = (\boldsymbol{Z}'\boldsymbol{Z})^{-1}\boldsymbol{Z}'\boldsymbol{X}$ [2]. Thus we proved the expression in Equation 7 is the BLUP for $\boldsymbol{\beta}$.

S.2 Simulation Results

We simulate data based on ideas implemented in [1]. For each simulation setting, we generate 100 replicates with $n = 100$ subjects in each dataset. We assume a balanced design with $n_i = 3$ visits for each subject and the time variable T_{ij} is generated by standardizing the visits (j=1,2,3) to have unit variance. $M = 300$ is the total number of observations in each simulation replicate. The functional curves $X_{ij}(t)$ with length of 600 are generated as follows,

$$\begin{cases} X_{ij}(t) &= \sum_{l=1}^{N_U} \xi_{il}\phi_l^{(U_0)}(t) + \sum_{l=1}^{N_U} T_{ij}\xi_{il}\phi_l^{(U_1)}(t) + \sum_{m=1}^{N_V} \zeta_{ijm}\phi_m^{(2)}(t), t \in \mathcal{D} \\ \xi_{il} &\overset{\text{i.i.d.}}{\sim} \mathcal{N}(0.\lambda_l^U), \zeta_{ijm} \overset{\text{i.i.d.}}{\sim} \mathcal{N}(0.\lambda_m^V) \end{cases}.$$

where the number of eigenfunctions $N_U = N_V = 4$ and the scores ξ_{il}'s and ζ_{ijm}'s are mutually independent. The eigenfunctions bases are set as,

$$\begin{array}{lll}\phi_1^{U_0}(t) = \sin(2\pi t), & \phi_1^{U_1}(t) = 1/2, & \phi_1^V(t) = 1 \\ \phi_2^{U_0}(t) = \cos(2\pi t), & \phi_2^{U_1}(t) = \sin(6\pi t), & \phi_2^V(t) = \sqrt{3}(2t-1) \\ \phi_3^{U_0}(t) = \sin(4\pi t), & \phi_3^{U_1}(t) = \cos(6\pi t), & \phi_3^V(t) = \sqrt{5}(6d^2 - 6d + 1) \\ \phi_4^{U_0}(t) = \cos(4\pi t), & \phi_4^{U_1}(t) = \sin(8\pi t), & \phi_4^V(t) = \sqrt{7}(20d^3 - 30d^2 + 12d - 1),\end{array}$$

where $\phi_l^{U_0}$ and $\phi_l^{U_1}$ are orthogonal but they are correlated with ϕ_m^V if $m \neq 1$.

The true eigenvalues have two scenarios,

1. level 1: $\lambda_1^U = 4, \lambda_l^U = 0.5^{l-1}, l = 2, 3, 4$ and level 2: $\lambda_m^V = 0.5^{m-1}, m = 1, 2, 3, 4$.
2. level 1: $\lambda_l^U = 0.5^{l-1}, l = 1, 2, 3, 4$ and level 2: $\lambda_1^V = 4, \lambda_m^V = 0.5^{m-1}, m = 2, 3, 4$.

For each of the 100 simulated datasets, we implemented the longitudinal FPCA, as described in section 3.2 to estimate the eigenfunctions, eigenvalues, scores and predicted functional trajectories. As a first assessment of model estimation accuracy, we computed the normalized errors between the estimated and true values of subject-level $(\hat{\xi}_{il} - \xi_{il})/\sqrt{\lambda_l^U}$ and visit-level $(\hat{\zeta}_{ijm} - \zeta_{ijm})/\sqrt{\lambda_m^V}$ scores. The results are displayed in S.1, which show that the score parameters are unbiasedly estimated. The simulation results demonstrate the agreement with simulation results in Greven et al. (2010)[1], which provided a more complete list of simulation examples.

As a second assessment of accuracy, we calculated three ways of *residual* $(R_{ij}(t))$ MSE described in section 3.2, which is defined as the total mean squared count difference per observation between the predicted and observed activity curves, i.e. $\frac{1}{M}\sum_{i,j}(\sum_t \mathsf{R\_\{ij\}(t)})^2$. The results are displayed in S.2, and the findings are discussed in section 3.2.

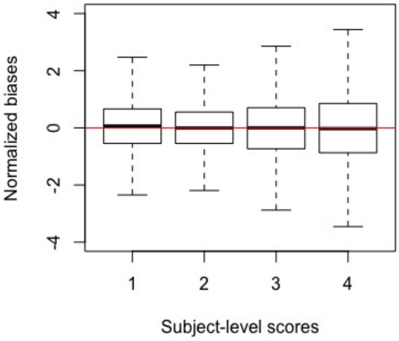

(a)

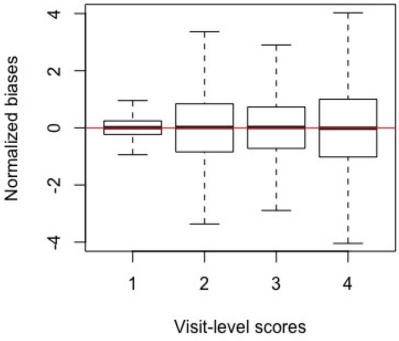

(b)

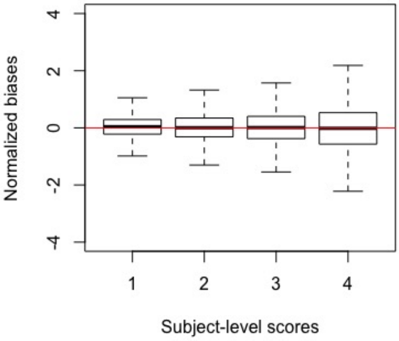

(c)

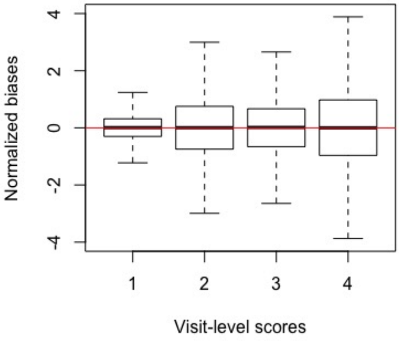

(d)

S.1. Boxplots of the normalized biases of estimated principal component scores for subject-level process $(\hat{\xi}_{il} - \xi_{il})/\sqrt{\lambda_l^U}$ (left) and visit-level $(\hat{\zeta}_{ijm} - \zeta_{ijm})/\sqrt{\lambda_m^V}$ (right) based on simulation scenario 1 (top) and scenario 2 (bottom) with 100 replicates. Red line represents the zero.

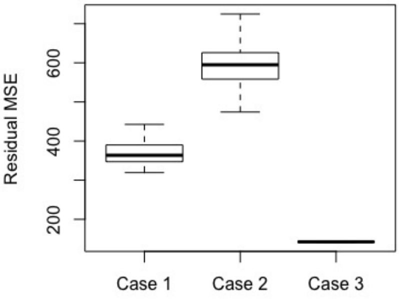

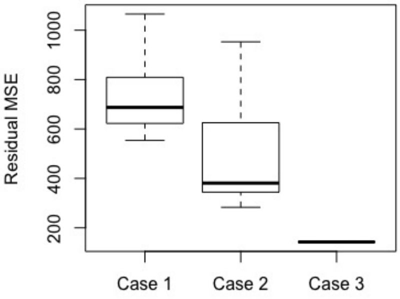

(a) **(b)**

S.2. Boxplots of residual MSE from stepwise decomposition based on simulation scenario 1 (a) and scenario 2 (b) with 100 replicates. From left to right, the mean squared errors are acquired from three ways of computing residuals: $X_{ij}(t) - U_i(t)$ (Case 1), $X_{ij}(t) - V_{ij}(t)$ (Case 2), $X_{ij}(t) - U_i(t) - V_{ij}(t)$ (Case 3)

S.3 Sensitivity Analysis

To assess, if averaging daily records impacted on our findings, we conducted three sensitivity analyses. In particular, we took (i) a random day, (ii) the first three-day average and the (iii) up to the first seven-day average for each participant at each visit, and then applied the same longitudinal FPCA on these new inputs. The results are provided S.1, S.2 and S.3. Compared with results in the original manuscript (Table 2 and S.4), averaging over daily inputs does not meaningfully alter results of either functional PCA or regression procedures. Specifically, although the variation explained by level-1 PCs is lower when only considering a random day, the overall variance explained by both level 1 and level 2 PCs are similar in general. Also, in the regression models, while point estimates vary, the findings are consistent across approaches, namely that higher person-level PC scores 1 and 2 are associated with lower (log)insulin and BMI. In fact, the averaged inputs could reduce the influence of random movements or activity on a single day, thus reducing noise in the data.

S.1. Percentages of average variance explained by different levels of principal components and regression results of log(insulin) and BMI on the first two level 1 and level 2 principal component scores using a-random-day data.

# Component	$\phi_l^{U_0}$	$\phi_l^{U_1}$	ϕ_m^2	cumulative variation explained
1	0.0764	0.0120	0.2543	0.3427
2	0.0200	0.0034	0.1606	0.5267
3	0.0125	0.0017	0.0836	0.6245
	0.1089	0.0171	0.4985	0.6245
Outcome	Predictor	Coefficient	SE	Confidence interval
Log(insulin)	PC11	-0.09	0.03	(-0.15, -0.04)
	PC12	-0.07	0.03	(-0.12, -0.01)
	PC21	-0.04	0.02	(-0.07, -0.01)
	PC22	0.00	0.01	(-0.03, 0.03)
BMI	PC11	-0.45	0.22	(-0.87, -0.03)
	PC12	-0.39	0.22	(-0.82, 0.04)
	PC21	-0.10	0.08	(-0.25, 0.05)
	PC22	0.10	0.07	(-0.03, 0.24)

S.2. Percentages of average variance explained by different levels of principal components and regression results of log(insulin) and BMI on the first two level 1 and level 2 principal component scores using three-day average data.

# Component	$\phi_l^{U_0}$	$\phi_l^{U_1}$	ϕ_m^2	cumulative variation explained
1	0.1507	0.0104	0.2160	0.3771
2	0.0391	0.0142	0.1322	0.5626
3	0.0206	0.0052	0.0898	0.6782
4	0.0152	0.0013	0.0585	0.7532
	0.2256	0.0311	0.4965	0.7532
Outcome	Predictor	Coefficient	SE	Confidence interval
Log(insulin)	PC11	-0.11	0.03	(-0.16, -0.05)
	PC12	-0.10	0.02	(-0.14, -0.06)
	PC21	-0.03	0.02	(-0.06, 0.00)
	PC22	0.01	0.02	(-0.02, 0.04)
BMI	PC11	-0.86	0.21	(-1.28, -0.44)
	PC12	-0.12	0.18	(-0.47, 0.22)
	PC21	-0.13	0.08	(-0.28, 0.02)
	PC22	0.13	0.02	(-0.12, 0.03)

S.3. Percentages of average variance explained by different levels of principal components and regression results of log(insulin) and BMI on the first two level 1 and level 2 principal component scores using up to seven-day average data.

# Component	$\phi_l^{U_0}$	$\phi_l^{U_1}$	ϕ_m^2	cumulative variation explained
1	0.2302	0.0220	0.1551	0.4073
2	0.0852	0.0167	0.1030	0.6122
3	0.0363	0.0044	0.0843	0.7372
4	0.0192	0.0013	0.0484	0.8061
	0.3709	0.0444	0.3908	0.8061
Outcome	Predictor	Coefficient	SE	Confidence interval
Log(insulin)	PC11	-0.14	0.04	(-0.22, -0.06)
	PC12	-0.09	0.03	(-0.15, -0.03)
	PC21	-0.13	0.04	(-0.20, -0.06)
	PC22	0.02	0.03	(-0.03, 0.08)
BMI	PC11	-0.78	0.29	(-1.34, -0.22)
	PC12	-0.39	0.24	(-0.86, 0.07)
	PC21	-0.19	0.19	(-0.56, 0.18)
	PC22	0.00	0.14	(-0.26, 0.27)

S.4 Examples of Stepwise Decomposition

Figure S.3 provides two examples from our dataset of step-wise reconstruction of the activity curves, after the eigen-decomposition. The first individual example has a large first level 1 principal component score but a small first level 2 principal component score, and vice versa for the second individual example. It further illustrates that principal component scores can inform explained variation at subject- and visit-level. Specifically, minimal between-visit difference is witnessed in the first example, i.e., adding in the visit-level component to the subject-level curves does not improve the fit materially, indicating the majority of variation is explained at subject-level. While in the second example which has a larger level 2 principal component score, the figure presents more significant variation between the two visits, and thus the visit-level component is needed to recapitulate the trends of the original data.

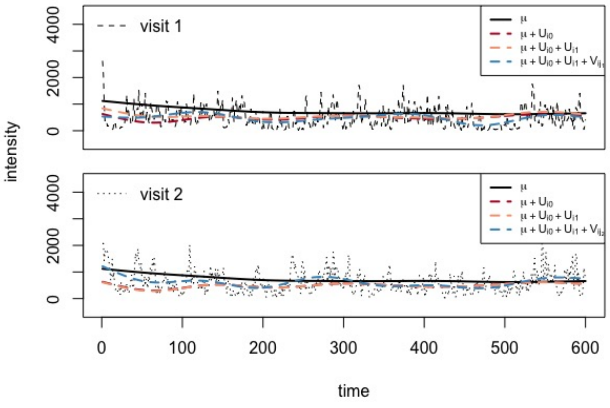

(a)

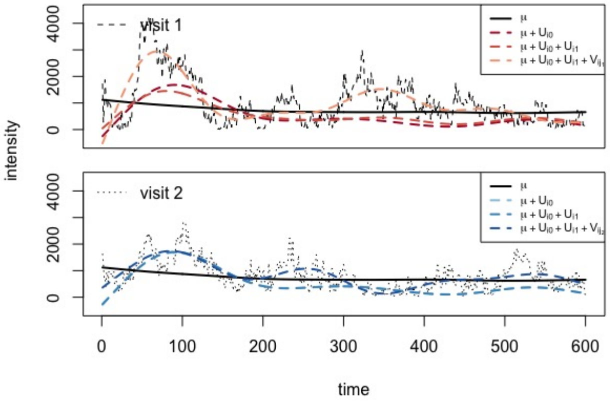

(b)

S.3. Stepwise decomposition of two examples of PA records with raw count inputs (black) and estimated curves at each visit (red, blue): (a) is an example with a large first level 1 principal component score but a small first level 2 principal component score; (b) is an example with a small first level 1 principal component score but a large first level 2 principal component score.

S.5 Additional Results

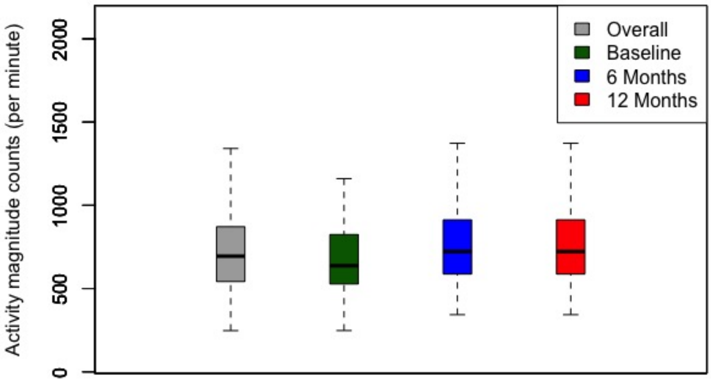

S.4. Boxplot of individual daily-average activity magnitude (sum of activity magnitudes over 600 minutes divided by 600) overall and at baseline, 6 months, 12 months. It illustrates an increase in PA magnitudes after baseline visits.

S.4. Percentages of average variance explained by different levels of principal components. The cumulative variation is the sum of row entries for the current row. The last row presents the cumulative variance for each column.

# Component	$\phi_l^{U_0}$	$\phi_l^{U_1}$	$\phi_m^{(2)}$	**cumulative variation explained**
1	0.2301	0.0228	0.2530	0.5059
2	0.0863	0.0163	0.1026	0.7111
3	0.0365	0.0048	0.0412	0.7935
4	0.0196	0.0015	0.0210	0.8356
5	0.0131	0.0022	0.0153	0.8662
	0.3855	0.0476	0.4331	0.8662

S.5. Linear mixed effect regression results of health outcomes on scaled total activity counts and MVPA respectively. It presents that both total activity counts and MVPA both exhibit a negative association with health outcomes, which supports the PCR results.

Outcome	Total Activity Counts	MVPA
Log(insulin)	-0.13 (-0.17, -0.09)	-0.12 (-0.16,-0.08)
Log(CRP)	-0.09 (-0.17, -0.01)	-0.09 (-0.17,-0.02)
BMI	-0.69 (-0.9, -0.48)	-0.63 (-0.84,-0.43)

*Adjusted for baseline age, ethnicity, smoking history and visit indicator.

References

[1] Greven S, Crainiceanu C, Caffo B et al. Longitudinal functional principal component analysis. In *Recent Advances in Functional Data Analysis and Related Topics*. Springer, 2011. pp. 149–154.

[2] Zipunnikov, Vadim and Greven, Sonja and Shou, Haochang and Caffo, Brian and Reich, Daniel S and Crainiceanu, Ciprian. Longitudinal high-dimensional principal components analysis with application to diffusion tensor imaging of multiple sclerosis. *The annals of applied statistics* 2014; 8(4): 2175.
